# Supplementary material for: Not all brawn, but some brain. Strength gains after training alters kinematic motor abundance in hopping
Source: PeerJ. 2018 Nov 23;6:e6010. doi: 10.7717/peerj.6010 (PMC6254240; doi:10.7717/peerj.6010)
Supplement: Table S1 [file peerj-06-6010-s001.docx]

**Supplemental**

**Table S1** Bi-weekly averaged exercise prescription for an individual training session

| **Weeks** |  | **1-2** | **3-4** | **5-6** |
| --- | --- | --- | --- | --- |
| *Conventional (3 sessions per week) – 18 supervised sessions total* | | | | |
| **Leg press** | Time based criterion for load increment of weekly adjusted 1RM | 2-3 sets x reps x 10 RM | 2-4 sets x 6 reps x 8RM | 2-4 sets x 4 reps x 6 RM |
| **Calf raise** |  | 2-3 sets x reps x 10 RM | 2-3 sets x 6 reps x 8RM | 2-4 sets x 4 reps x 6 RM |
| **Lunge** |  | 2-3 sets x reps x 10 RM | 2-3 sets x 6 reps x 8RM | 2-4 sets x 4 reps x 6 RM |
| *Load carriage specific (3 sessions per week)- 18 supervised sessions total* | | | | |
| **SL hopping** | SL hopping and CMJ time based criterion for load increment.  Hip flexor pull time based criterion for load increment of weekly adjusted 1RM | 2-4 sets x 20 s x 2.2 Hz x 110% BW | 2-4 sets x 20 s x 2.2 Hz x 115 - 120% BW | 2-4 sets x 20 s x 3 Hz x 120% BW |
| **CMJ** |  | 5-10 sets x 2-3 reps x 100-105% BW | 5-10 sets x 2-3 reps x 110-115% BW | 5-10 sets x 2-3 reps x 120% BW |
| **Hip flexor pull** |  | 8-10 sets x 2-3 reps x 10RM | 8-10 sets x 2-3 reps x 8RM | 8-10 sets x 2-3 reps x 6RM |
| Abbreviations: reps = repetitions; Hz = Hertz; SL = single leg; CMJ = countermovement jump; RM = repetition maximum; BW = body weight | | | | |
